# Role of Steroids in Sickle Cell Patients With Acute Chest Syndrome

**DOI:** 10.7759/cureus.26196

**Published:** 2022-06-22

**Authors:** Kokila Jeyamurugan, Min-Kyung Jung, Fernanda E Kupferman, Kusum Viswanathan

**Affiliations:** 1 Pediatrics, Brookdale University Hospital Medical Center, Brooklyn, USA; 2 Statistics, New York Institute of Technology College of Osteopathic Medicine, Old Westbury, USA

**Keywords:** acute chest syndrome, asthma, bronchodilators, sickle cell disease, corticosteroids

## Abstract

Background

The role of corticosteroids to treat acute chest syndrome (ACS) in patients with sickle cell disease (SCD) has always been a matter of debate. In clinical practice, systemic steroids were given for ACS with more severe disease. With the lack of standard treatment guidelines, their use to treat ACS is highly physician-dependent and varies widely across different hospitals. The utility of corticosteroids in ACS remains unclear. The objective of our study was to describe the differences between SCD patients treated with corticosteroids for ACS and those who were not and to evaluate the association between corticosteroid use, length of stay, and readmission rates.

Methodology

We performed a retrospective chart review of patients with SCD ≤18 years of age hospitalized for ACS at Brookdale University Hospital Medical Center between January 2016 and May 2021.

Results

We identified 43 patients with 60 episodes of ACS (median age was 11 years and 55% were males). In total, 32 such episodes were treated with corticosteroids. The use of bronchodilators (p = 0.23), hydroxyurea (p = 0.13), and the presence of fever (p = 0.86) showed no significant difference between the two groups. The need for blood transfusions (p = 0.005), intensive care unit admission (p = 0.031), respiratory support (p = 0.011), and chest X-ray finding with more than one lobe involvement (p = 0.003) all point to moderate or severe ACS, which has been linked to steroid use. The length of hospital stay (p = 0.07) and the readmission rate (p = 0.31) were not statistically significant between the groups. Even in the subgroup with asthma, the length of stay was not different between the groups (p = 0.44).

Conclusions

Our results show that treatment with systemic steroids for ACS is associated with more severe disease. The length of hospital stay was not different between the steroid-treated and untreated groups. Corticosteroids were not associated with a higher readmission rate in our study population, even in ACS patients with comorbid asthma. Further adequately powered prospective trials are needed to investigate the efficacy of corticosteroids in ACS.

## Introduction

Sickle cell disease (SCD) is a group of inherited red blood disorders caused by a single-point mutation in the gene encoding for the β-globin chain of hemoglobin (Hb) [1]. It is estimated that more than two million US residents are either heterozygous or homozygous for genetic substitution. The majority of those affected are of African origin and a minority are of Hispanic or Southern European, Middle Eastern, or Asian Indian descent [2]. The most prevalent types of SCD include homozygous hemoglobin SS (HbSS) and the compound heterozygous conditions such as hemoglobin Sβ0-thalassemia (HbSβ0-thalassemia), hemoglobin S β+-thalassemia (HbS β+-thalassemia), and hemoglobin sickle cell disease (HbSC) [2].

Acute chest syndrome (ACS) is one of the most frequent and serious acute complications of SCD [2]. Data from the Cooperative Study of Sickle Cell Disease (CSSCD) suggest that approximately 50% of children with HbSS disease experience at least one episode of ACS by 10 years of age [3]. After a painful crisis, ACS is the second most prevalent reason for hospitalization in people with SCD and is the most common cause of premature death [4]. Clinically, ACS resembles pneumonia and can develop suddenly or insidiously during hospitalization for another complication of SCD, most frequently vaso-occlusive crises [2].

ACS is typically defined as the appearance of new pulmonary infiltrates on chest imaging involving at least one complete lung segment accompanied by respiratory signs or symptoms, such as chest pain, cough, wheeze, tachypnea, hypoxemia, or fever [1]. The clinical manifestations of ACS may be subtle in the early stages [2]. The most common etiology of ACS is infection but the complication may also result from a pulmonary fat embolism, infarction, atelectasis, or pulmonary edema [2,5]. In many cases, the specific cause or inciting factor is often unknown. There are no distinctive laboratory characteristics of ACS, although the Hb concentration often falls sharply below the patient’s steady-state value [2].

Management of ACS remains mostly supportive and empiric and may include broad-spectrum antibiotics, supplemental oxygen, pain medications, bronchodilators, and blood transfusions [2,5]. Markers of an impending severe ACS are multilobe disease, increased work of breathing, hypoxia with an inability to maintain oxygen saturation above 95% even with supplemental oxygen, and pleural effusions [2]. It has been reported that inflammation plays an important role in the development of ACS, and, as such, corticosteroids have been studied as a potential targeted treatment [5]. A study performed on 43 children with mild-to-moderate ACS found a 40% reduction in the length of hospitalization in patients treated with dexamethasone; however, treated patients had a higher readmission rate within 72 hours mostly for vaso-occlusive pain crisis [6]. A brief course of prednisolone was shown to significantly attenuate the course of ACS, but the treatment group appeared to have an increased risk of readmission, generally for pain episodes, although the re-admission rate was not significantly greater compared to controls [7]. In a retrospective study involving 31 patients with severe ACS, treatment with both corticosteroids and transfusion showed no significant risk of increased readmissions for vaso-occlusive crises [8]. However, in another retrospective database study, treatment with corticosteroids was associated with an increased length of hospital stay and a higher three-day readmission rate. There was a significant variation in the use of corticosteroids for ACS among the different hospitals that participated in the study [5].

In clinical practice, systemic corticosteroids were given for ACS with more severe disease. However, over the years, corticosteroids have become a less popular treatment choice with concern over side effects and toxicities associated with their use [5]. Considering the conflicting data in the literature and the lack of standard treatment guidelines, more research is needed to investigate the efficacy of corticosteroid use in sickle cell patients with ACS.

Our primary aim was to describe the differences between patients treated with corticosteroids for ACS and those who were not and to evaluate the association between corticosteroid use and length of hospital stay and readmission rates. We hypothesized that the administration of corticosteroids during ACS would be associated with a decrease in the length of hospital stay and would lower the risk of readmission.

This article was previously presented as a meeting abstract at the 2022 Pediatric Academic Societies (PAS) meeting on April 22, 2022.

## Materials and methods

Data for this retrospective analysis were collected from our hospital’s electronic medical records. The study protocol was approved by the Institutional Review Board at Brookdale University Hospital Medical Center (approval number: OBH-B-21-38).

We included all patients with SCD with one of four SCD genotypes (SS, SC, S-beta+-thalassemia, and S-beta0-thalassemia) aged ≤18 years and of both sexes, who had an episode of ACS and were admitted to the pediatric inpatient unit at Brookdale Hospital Medical Center between January 2016 and May 2021.

We identified eligible patients based on the discharge diagnoses of SCD and ACS using the appropriate International Statistical Classification of Diseases 10th Revision (ICD-10) codes. Diagnosis of asthma was defined if a history of asthma was recorded in the patient chart. A temperature of ≥38.5°C was considered a fever. Readmission was defined as hospitalization for sickle crises within 14 days of discharge. Duration of ACS was the time from the diagnosis of ACS to either discharge or resolution of symptoms. Children with underlying immunodeficiency, recent positive blood culture, and on inhalational corticosteroids were excluded.

The unit of analysis was each distinct hospitalization for an episode of ACS. Patients were organized into two groups based on corticosteroid use to treat ACS. The primary outcome measure was the length of hospital stay (in days) and readmission for sickle crisis within 14 days of hospital discharge. To adjust for confounding, multiple other variables that could help define disease severity were identified. These included demographic data (age, sex), additional therapies likely to be used such as the use of bronchodilators, hydroxyurea, parenteral pain medications, oxygen requirement, need for intensive care, respiratory support, and blood transfusions. Other markers of severity such as radiological findings of more than one lung lobe involvement, any prior ACS episodes, or ≥three hospitalizations for sickle cell crises in the preceding 12 months were included. We performed subgroup analysis in patients with and without comorbid asthma to determine whether the association between corticosteroid use and length of hospital stay was different in these groups.

To describe demographic data, the mean and standard deviation was computed for continuous variables and the frequency and percentage for categorical variables. To test the differences between patients treated with corticosteroids for ACS and those who were not the Mann-Whitney U test was used for continuous outcome variables after checking for normal distribution and equal variances and the chi-square test for categorical outcome variables. A linear regression model was fitted to data to study the association between corticosteroid use and the length of hospital stay, and a logistic regression model to study the association between corticosteroid use and the readmission rate. Statistical significance was evaluated with α=0.05. SPSS Version 26 (IBM Corp., Armonk, NY, USA) was used for all statistical analyses.

## Results

We identified 43 patients with 60 episodes of ACS. Among them, nine patients had multiple hospitalizations for ACS. The median age of the cohort was 11 years (range = 2-18 years). ACS episodes were more common in males than females (55% vs. 45%). Out of 60 episodes, 32 (53.3%) were treated with corticosteroids. Bronchodilators were used in 47 (78.3%) episodes and hydroxyurea in 46 (76.7%) episodes. Fever was identified in 40 (66.7%) episodes. Overall, 31 (51.7%) episodes were associated with asthma. The use of bronchodilators, hydroxyurea, and the presence of fever showed no significant difference between the corticosteroid-treated and untreated groups (Table 1).

**Table 1:** Comparison of categorical variables among the corticosteroid-treated and untreated groups. ACS: acute chest syndrome; ICU: intensive care unit; SCD: sickle cell disease

Variable (Categorical)	Corticosteroids (N = 32)	No corticosteroids (N = 28)	Total (N = 60)	P-value
	Count (Percentage)	Count (Percentage)	Count (Percentage)	
Sex				0.08
Male	21 (65.6%)	12 (42.9%)	33 (55.0%)	
Female	11 (34.4%)	16 (57.1%)	27 (45.0%)	
Bronchodilator use				0.23
Yes	27 (84.4%)	20 (71.4%)	47 (78.3%)	
No	5 (15.6%)	8 (28.6%)	13 (21.7%)	
Fever ≥38.5°C				0.86
Yes	21 (65.6%)	19 (67.9%)	40 (66.7%)	
No	11 (34.4%)	9 (32.1%)	20 (33.3%)	
Hydroxyurea				0.13
Yes	27 (84.4%)	19 (67.9%)	46 (76.7%)	
No	5 (15.6%)	9 (32.1%)	14 (23.3%)	
Asthma				0.07
Yes	20 (62.5%)	11 (39.3%)	31 (51.7%)	
No	12 (37.5%)	17 (60.7%)	29 (48.3%)	
Prior ACS episodes in the last 12 months				0.12
0	17 (53.1%)	22 (78.6%)	39 (65.0%)	
1	12 (37.5%)	5 (17.9%)	17 (28.3%)	
2	3 (9.4%)	1 (3.6%)	4 (6.7%)	
Length of hospital stay (day)				0.07
3–5 days	12 (37.5%)	17 (60.7%)	29 (48.3%)	
6 days or longer	20 (62.5%)	11 (39.3%)	31 (51.7%)	
Blood transfusion				0.005
Yes	29 (93.5%)	18 (64.3%)	47 (79.7%)	
No	2 (6.5%)	10 (35.7%)	12 (20.3%)	
High dependency/ICU admission				0.031
Yes	14 (43.8%)	5 (17.9%)	19 (31.7%)	
No	18 (56.3%)	23 (82.1%)	41 (68.3%)	
Readmission within 14 days of discharge				0.31
Yes	5 (15.6%)	2 (7.1%)	7 (11.7%)	
No	27 (84.4%)	26 (92.9%)	53 (88.3%)	
Multiple SCD admissions in the last 12 months				0.34
Yes	12 (37.5%)	7 (25.9%)	19 (32.2%)	
No	20 (62.5%)	20 (74.1%)	40 (67.8%)	
Respiratory support				0.011
Yes	9 (28.1%)	1 (3.6%)	10 (16.7%)	
No	23 (71.9%)	27 (96.4%)	50 (83.3%)	
Chest X-ray findings (number of lobes involved)				0.003
Less than 1	0 (0.0%)	3 (10.7%)	3 (5.0%)	
1	17 (53.1%)	22 (78.6%)	39 (65.0%)	
More than 1	15 (46.9%)	3 (10.7%)	18 (30.0%)	

Both groups were similar with regards to the duration of ACS (p = 0.4), fetal hemoglobin percentage (HbF%) (p = 0.14), oxygen saturation at the time of admission (p = 0.99) and during hospital stay (p = 0.47), use of parenteral pain medication (p = 0.4), and oxygen requirement (p = 0.6) (Table 2).

**Table 2:** Comparison of continuous variables among the corticosteroid-treated and untreated groups. ACS: acute chest syndrome; HbF%: fetal hemoglobin percentage

Variable (Continuous)	Corticosteroids (N = 32)	No corticosteroids (N = 28)	Total (N = 60)	P-value
	Median (Min, Max)	Median (Min, Max)	Median (Min, Max)	
Age (year)	11 (2, 18)	11 (2, 18)	11 (2, 18)	0.41
Duration of ACS (day)	4 (2, 10)	4 (2, 9)	4 (2, 10)	0.4
Oxygen saturation in room air on admission (%)	98 (88, 100)	98 (79, 100)	98 (79, 100)	0.99
Lowest oxygen saturation in room air during hospital stay (%)	94 (80, 98)	95 (77, 98)	95 (77, 98)	0.47
HbF%	11.5 (0.0, 20.4)	13 (0.3, 82.2)	12.1 (0.0, 82.2)	0.14
Duration of oxygen requirement (day)	1 (0, 5)	0 (0, 9)	1 (0, 9)	0.69
Duration of intravenous pain medication (day)	3 (0, 8)	2 (0, 12)	2 (0, 12)	0.4
Duration of respiratory support (day)	0 (0, 8)	0 (0, 1)	0 (0, 8)	0.019

The length of hospital stay varied between three and five days for 29 (48.3%) episodes. For prolonged hospitalizations of six days and longer, the length of stay was more in the corticosteroid-treated group (20 vs. 11 in control); however, the difference was not clinically significant (p = 0.07). Patients were readmitted for pain crises within 14 days of hospital discharge after seven (11.7%) episodes, although the difference between the groups (15.6% vs. 7.1%) did not reach statistical significance (Table 3).

**Table 3:** Comparison of length of hospital stay and readmission rate by corticosteroid treatment.

	Corticosteroids (N = 32)	No corticosteroids (N = 28)	P-value
	Count (Percent)	Count (Percentage)	
Length of hospital stay			0.07
3–5 days	12 (37.5%)	17 (60.7%)	
6 days or longer	20 (62.5%)	11 (39.3%)	
Readmission within 14 days of discharge			0.31
3–5 days	5 (15.6%)	2 (7.1%)	
26 days or longer	27 (84.4%)	26 (92.9%)	

The number of prior ACS episodes (p = 0.12) and SCD admissions (p = 0.34) in the preceding 12 months was not different between the comparison groups. Overall, 47 (79.7%) episodes required blood transfusions, of which 29 (93.5%) were in the steroid-treated group (p = 0.005). Of the total 60 hospitalizations, 19 required ICU care (14 vs. 5; p = 0.031), with 10 (9 vs. 1, p = 0.011) requiring respiratory support, mostly noninvasive ventilation. Chest X-ray findings of ≥one lobe involvement indicating severe disease was often seen in the corticosteroid-treated group (p = 0.003).

**Figure 1:**
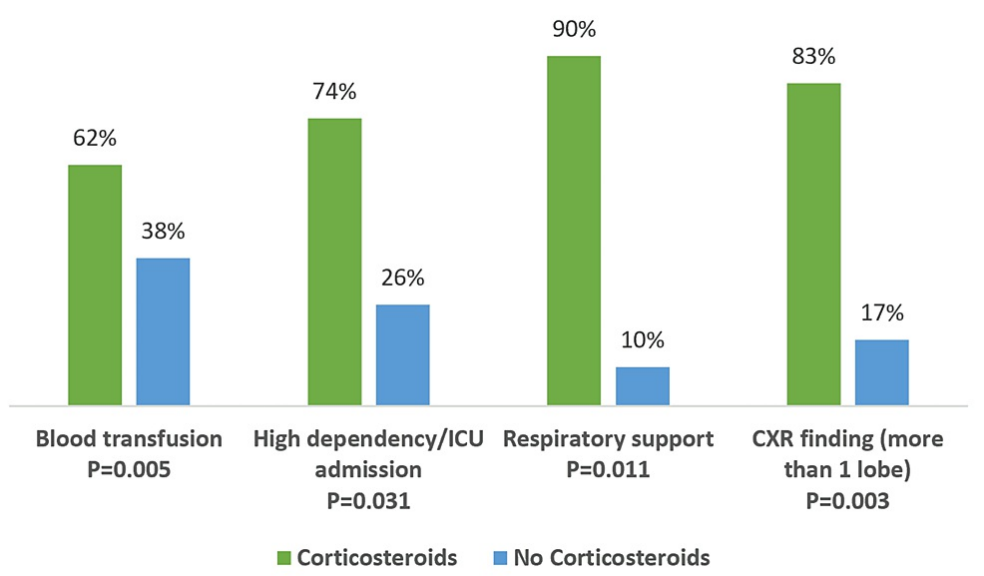
Comparison of variables related to the severity of ACS. ACS: acute chest syndrome; CXR: chest X-ray; ICU: intensive care unit

The need for blood transfusions, ICU admission with respiratory support, and a chest X-ray finding with more than one lobe involvement all point to moderate or severe ACS, which has been linked to steroid use (Figure 1).

On further analyzing the association of asthma with corticosteroid use and length of hospital stay, the length of stay was not found to be statistically significant (p = 0.44) between the groups (Table 4).

**Table 4:** Association of asthma with corticosteroid use and length of hospital stay.

Length of hospital stay	Corticosteroids (N = 32)	No corticosteroids (N = 28)	P-value
	Count (Percentage)	Count (Percentage)	
Asthma			0.44
3–5 days	8 (40.0%)	6 (54.5%)	
6 days or longer	12 (60.0%)	5 (45.5%)	
No asthma			0.1
3–5 days	4 (33.3%)	11 (64.7%)	
26 days or longer	8 (66.7%)	6 (35.3%)	

## Discussion

ACS is a major cause of morbidity and mortality in patients with SCD [5,9]. After the pain crisis, ACS is the second leading cause of hospitalization [10,11]. The decision to administer steroids for the treatment of ACS varies widely based on the severity of the clinical presentation [5].

Our study showed that ACS episodes were more in males than females which is similar to the previous observation by Alkindi et al. and Nansseu et al. [12,13]. It has been reported that females with SCD tend to have higher HbF levels which may offer some protection against ACS by increasing the bioavailability of nitric oxide, although this was not analyzed in our study [14]. HbF%, oxygen saturation, and fever were similar in both the steroid-exposed and unexposed groups, although previous studies have shown a lower HbF%, hypoxia, and fever to be significant predictors for severe ACS [9,12,15].

The use of non-invasive ventilation (NIV) and simple/exchange transfusions are markers of serious illness [12]. In our study, corticosteroid use was associated with multiple measures of severity, including ICU admission and the requirement of respiratory support. A prospective study from a single institution demonstrated that NIV improved respiratory rate and gas exchange in ACS compared to oxygen alone. However, NIV did not significantly reduce the number of patients remaining hypoxemic on day three. No differences were noted in pain relief, blood transfusion requirements, or length of stay [16]. The majority of our patients with ACS required blood transfusion, more in the steroid-treated group compared to controls, which is in line with what is known from previous studies [12,15]. In a study involving 36 children with SCD, patients who required transfusion had more severe disease on admission; however, the study demonstrated no significant difference in the duration of fever or hospital stay between the transfusion and the non-transfusion groups [17].

The number of previous admissions for SCD and prior ACS episodes was found to be higher in the corticosteroid-treated group, albeit they did not reach statistical significance. A history of previous hospital admission remains an independent risk factor for ACS [18]. This has also been proven in a large retrospective study by Quinn et al. [19,20]. In the absence of optimal treatment guidelines, the decision to administer steroids is highly dependent on the clinical decision of the treatment team. It is likely that the history of previous hospitalizations may have influenced steroid use as patients may have been deemed to be potentially more severe. In our study, the duration of ACS was similar in both groups with a median duration of four days (range = 2-10), raising the possibility that the administration of steroids helped in quicker recovery.

Asthma is a common comorbidity in patients with SCD. ACS occurs with increased frequency in children with SCD who have asthma or prior ACS events [5,12]. In a cohort of infants followed by the CSSCD, patients with SCD with a concurrent diagnosis of asthma were reported to have more frequent ACS episodes (0.39 vs. 0.20 events per patient-year; p < 0.001) and painful episodes (1.39 vs. 0.47 events per patient-year; p < 0.001) [10,21]. When comparing children with asthma to those without asthma, no significant difference was reported in the length of hospital stay for ACS episodes [10]. In our analysis, however, it was clear that patients with asthma were more likely to receive steroids. There was no statistically significant difference in the length of stay between the corticosteroid-treated and untreated groups, suggesting that steroids may have played a role in reducing the length of stay in patients with asthma.

Interestingly, in our population, corticosteroid use was associated with a 23% increase in the prolonged length of hospital stay [20 (62.5%) vs. 11 (39.3%)]. However, the value did not reach statistical significance, and a larger sample may be necessary to see any correlation. Again, this suggests that patients who are deemed to be sicker are more likely to receive steroids. Although previous research has documented the efficacy of steroids in reducing the duration of ACS, the substantial risk of readmission for pain limits its use [22]. In our cohort, the use of steroids did not increase the readmission rates for the vaso-occlusive crisis which is in contrast to what is known from prior studies [5-8,22].

Our study has some limitations including a small sample size and being a retrospective cohort study based on a single institution. Given the nature of the dataset, baseline demographic characteristics, and disease severity, there is a possibility of unmeasured confounders even after adjusting for potential confounders.

## Conclusions

In summary, the utilization of steroids for the treatment of ACS is associated with more severe disease. Our study failed to show a significant difference in the length of hospital stay between the steroid-treated and untreated groups. There was no increased risk of readmission even in patients with comorbid asthma. Until further adequately powered prospective trials become available to investigate the benefits and risks of corticosteroid use in ACS, corticosteroids may continue to be used for sicker patients with severe disease at the discretion of the clinician.
